# Systemic Inflammation Response Index Is a Promising Prognostic Marker in Elderly Patients With Heart Failure: A Retrospective Cohort Study

**DOI:** 10.3389/fcvm.2022.871031

**Published:** 2022-07-14

**Authors:** Xue Wang, Qingwei Ni, Jie Wang, Shujie Wu, Peng Chen, Dawei Xing

**Affiliations:** Department of Cardiology, The Second Affiliated Hospital and Yuying Children's Hospital, Wenzhou Medical University, Wenzhou, China

**Keywords:** systemic inflammation response index, elderly, heart failure, mortality, inflammation

## Abstract

**Background:**

Heart failure (HF) is a clinical syndrome caused by ventricular dysfunction, which leads to the decline of activity tolerance and repeated hospitalization, which seriously affects the quality of life and is the main cause of death of the elderly. It has long been observed that the pathophysiological mechanism of HF is associated with systemic inflammation. This study aims to explore the association between the systemic inflammation response index (SIRI), a novel biomarker of inflammation, and outcomes in elderly patients with HF.

**Methods:**

Data was extracted from the Medical Information Mart data for Intensive Care III (MIMIC-III) database and the Second Affiliated Hospital of Wenzhou Medical University. The primary outcome was 90-day all-cause mortality. The secondary outcomes included 1-year all-cause mortality, the length of hospital or intensive care unit (ICU) stay, and the need for renal replacement therapy (RRT). Cox proportional hazards regression, linear regression, and logistic regression models were used to assess the association between SIRI levels and all-cause mortality, the length of hospital or ICU stay, the need for RRT, respectively. Moreover, Pearson correlation analysis was conducted to evaluate the correlation between SIRI and C-reactive protein (CRP).

**Results:**

This study cohort included 3,964 patients from the MIMIC-III database and 261 patients from the Second Affiliated Hospital of Wenzhou Medical University. The result suggested that SIRI was independently associated with the 90-day, and 1-year all-cause mortality in elderly patients with HF (tertile 3 vs. tertile 1: adjusted HR, 95% CI: 1.41 (1.18, 1.68), 1.19 (1.03, 1.37); *p* trend = 0.0013, 0.0260; respectively). Elevated SIRI was associated with increased the length of hospital or ICU stay after adjusting for multiple confounders (tertile 3 vs. tertile 1: *β*, 95% CI: 0.85 (0.16, 1.54); 0.62 (0.18, 1.06); *p* trend = 0.0095, 0.0046; respectively). Furthermore, we found that patients with higher SIRI levels were more likely to require RRT (tertile 3 vs. tertile 1: OR, 95% CI: 1.55 (1.06, 2.28); *p* trend = 0.0459). Moreover, we confirmed that SIRI was statistically positively correlated with CRP (correlation coefficient *r* = 0.343, *p* <0.001).

**Conclusions:**

SIRI could be a novel promising inflammatory biomarker for predicting all-cause mortality in elderly patients with HF. And the patients with higher SIRI values had the longer length of hospital or ICU stay and were more likely to require for RRT. Of note, this study also verified a statistically significant positive correlation between SIRI and the inflammatory marker CRP, highlighting the importance of systemic inflammation as a determinant of outcome in patients with HF.

## Introduction

Heart failure (HF) is a clinical syndrome with symptoms and/or signs caused by a structural and/or functional cardiac abnormality and corroborated by elevated natriuretic peptide levels and/or objective evidence of pulmonary or systemic congestion. In recent years, great progress has been made in the treatment of HF, but the prevalence, mortality and rehospitalization rate of elderly patients are still high. It is reported that HF is a leading cause of mortality in the elderly the incidence and prevalence of HF are higher in people aged over 60 years (1). As is known to all, elderly patients have many risk factors for cardiovascular disease, various comorbidities, multiple medication and other characteristics. Moreover, HF is associated with high medical-related costs, a huge burden to patients and society (2). However, with the increasing trend of population aging and the increase of cardiovascular disease and its risk factors, the prevalence of heart failure in the elderly is expected to continue to rise in the future. Therefore, exploring the prognostic indicators of elderly patients with HF can assist in medical decision-making and identification of high-risk patients.

Systemic inflammation has been recognized as a common pathophysiological feature of both acute and chronic HF. The innate and adaptive immune systems are activated in HF, comprising non-cellular and cellular components. Among them, inflammatory cells mainly include neutral granulocytes, monocycral macrophages, eosinophils, alkaline cells, hypertrophic cells, natural killing cells, lymphocytes (T cells and B cells), and cytokines released by them (such as interleukin-6 (IL-6), tumor necrosis factor (TNF), etc.). Leukocytes and their subtypes are classical biomarkers of inflammation in cardiovascular diseases (3). The incidence of acute decompensated heart failure (ADHF) in patients with acute myocardial infarction increased with the increase of neutrophils levels (4). Circulating monocytes in patients with chronic heart failure (CHF) increased and the levels of which was associated with the severity of the disease (5). Moreover, lymphopenia is also related with poor prognosis in patients with HF (6). Growing evidence suggests that various inflammatory cells have been shown to play an important role in HF and were predictors of poor prognosis of HF (7).

In recent years, composite inflammation indicators are of great significance to inflammation and are widely used in clinical practice, such as Neutrophil-to-Lymphocyte Ratio (NLR), and Monocyte-to-Lymphocyte Ratio (MLR) and Platelet-to-Lymphocyte Raito (PLR). These indicators are considered to be inexpensive and easily accessible biomarkers that are associated with increased risk of coronary artery disease (CAD) (8, 9), stroke (10), and overall death (11). Recently, a novel indicator has emerged called the systemic inflammatory response index (SIRI). A composite index based on the absolute count of three different inflammatory cells, namely, neutrophils, monocytes, and lymphocytes, and it is highly associated with cancer, hyperuricemia, rheumatoid arthritis, and stroke (12–15). However, the prognostic value of SIRI in elderly patients with HF remains unclear. Consequently, this study aimed to evaluate the relationship between SIRI and outcomes in elderly patients with HF.

As a reflection of inflammation, C-reactive protein (CRP) is the most widely studied and clinically applied inflammatory marker to date. A large body of evidence suggests that elevated CRP is associated with adverse outcomes in HF patients (16). Therefore, another object of this study was to explored the relationship between SIRI and CRP.

## Materials and Methods

### Study Population

Data were obtained from a publicly available MIMIC-III database ver. 1.4 and the Second Affiliated Hospital of Wenzhou Medical University. MIMIC-III database contains deidentified health data for >50,000 critical care patients who were admitted to the Beth Israel Deaconess Medical Center (Boston, MA, USA) from 2001 to 2012 (17). We had to completed the National Institutes of Health's web-based course before accessing the database. The MIMIC-III database used in this study was approved by the Institutional Review Boards of Massachusetts Institute of Technology and Beth Israel Deaconess Medical Center. Moreover, this study was approved by the ethics committee of the Second Affiliated Hospital of Wenzhou Medical University (Approval number: 2021-K-71-01). Informed consent was not required in this study as the data was collected anonymously.

HF patients were identified by the International Classification of Diseases, ninth revision (ICD-9) code. We only included data for patients aged ≥ 60 years in the first ICU or hospital admission. The exclusion criteria were: (1) length of ICU or hospital stay was <24 h, or (2) missing key data.

### Study Variables and Outcomes

Data covariates including age, gender, race, vital signs, laboratory characteristics, comorbidities, and scoring systems were collected. Vital signs included temperature, respiratory rate, heart rate, systolic blood pressure (SBP), diastolic blood pressure (DBP), mean arterial pressure (MAP), and oxygen saturation (SPO2). Comorbidities included atrial fibrillation, CAD, valvular disease, chronic obstructive pulmonary disease (COPD), respiratory failure, chronic liver disease, chronic kidney disease (CKD), peripheral vascular disease, sepsis, need for renal replacement therapy (RRT), diabetes mellitus (DM), pneumonia, hypertension, and system inflammatory response syndrome (SIRS). Moreover, laboratory characteristics included neutrophil count, monocyte count, lymphocyte count, SIRI, white blood cell count, hemoglobin, platelet count, red cell volume distribution width (RDW), serum glucose, serum creatinine, blood urea nitrogen (BUN), anion gap, cardiac troponin I (cTN-I), N terminal pro B type natriuretic peptide (NT-proBNP), and CRP over the first 24 h in the ICU or hospital and left ventricular ejection fraction (LVEF). The Sequential Organ Failure Assessment (SOFA) scores and the Simplified Acute Physiology Score II (SAPS II) were also included. The SIRI was defined as neutrophil count × monocyte count/lymphocyte count.

The primary outcome was the 90-day all-cause mortality. The secondary outcomes were 1-year all-cause mortality, length of hospital or ICU stay, and need for RRT.

### Statistical Analyses

Continuous variables were presented as the mean ± standard deviation (SD), and the Kruskal-Wallis test was used to determine differences between groups. Categorical variables were expressed as numbers and percentages. The Chi-square test or Fisher's exact test were used for inter-group comparisons. A two-sided *p* < 0.05 was considered statistically significant. All statistical analyses were performed using EmpowerStats software ver. 2.0 and R software ver. 4.0.3.

We first analyzed the association between SIRI levels and all-cause mortality. The SIRI values were divided into tertiles or quintiles, and the first tertile or quintile was selected as the reference group. Cox regression analysis and smooth curve fitting were used to evaluate the independent effects of SIRI levels on mortality. We adjusted for age, sex, ethnicity, systolic blood pressure, diastolic blood pressure, systemic inflammatory response syndrome, serum creatinine, hemoglobin, white blood cell count, platelet count, red blood cell volume distribution width, atrial fibrillation, coronary artery disease, chronic kidney disease, respiratory failure, pneumonia, and hypertension. These confounders were selected based on their associations with the outcome of interest or a change in effect estimate of more than 10%. Second, linear regression models were used to evaluate the relationship between the length of hospital or ICU stay and SIRI adjusting for age, sex, ethnicity, blood urea nitrogen, white blood cell count, platelet count, atrial fibrillation, respiratory failure, pneumonia, SOFA score, and SASP II score. The impact of SIRI levels on the need for RRT in elderly patients with HF was estimated using logistic regression model adjusting for age, sex, ethnicity, diastolic blood pressure, respiratory rate, anion gap, serum creatinine, white blood cell count, platelet count, red blood cell volume distribution width, atrial fibrillation, chronic kidney disease, respiratory failure, pneumonia, SOFA score, and SASP II score. Third, stratification analyses were performed to examine the effect of SIRI levels in different subgroups using various parameters and comorbidities. Moreover, in order to evaluate the ability of SIRI to predict 90-day all-cause mortality, the receiver operating characteristic (ROC) curve was plotted with specificity and sensitivity and the area under the curve (AUC) for different parametric models was measured and compared.

Pearson correlation analysis was used to verify the correlation between SIRI, neutrophils, monocytes, leukocytes and CRP, respectively. The correlation coefficients (r) were deemed statistically significant when the *p* value was <0.05.

## Results

### Patient Characteristics

**Figure 1:**
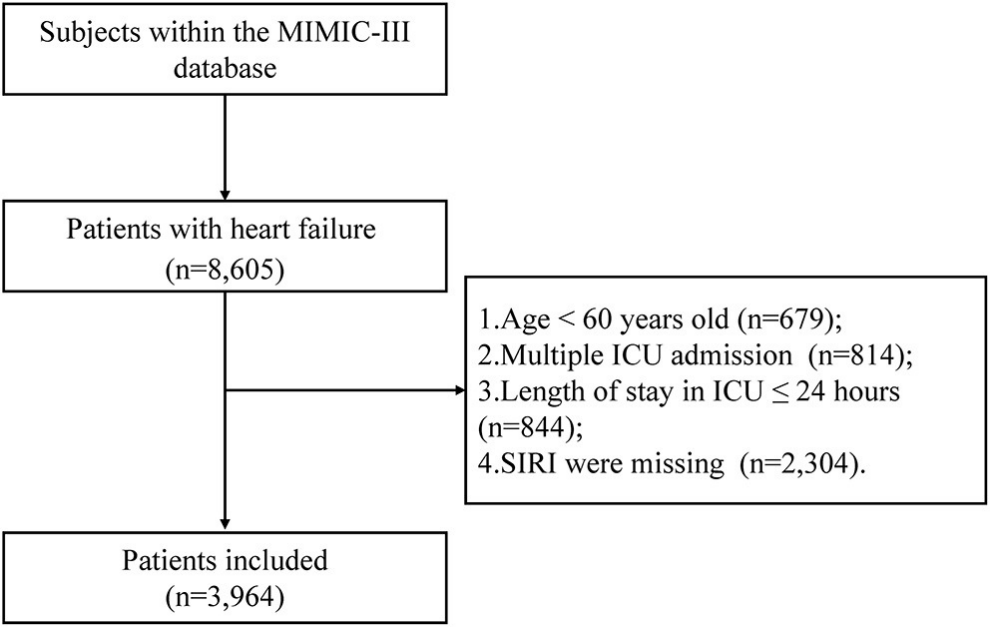
Flow chart of study population selection. Abbreviation: ICU: intensive care unit; MIMIC-III: Medical Information Mart for Intensive Care-III; SIRI: systemic inflammation response index, SIRI is calculated using the counts of peripheral venous blood neutrophils (N), monocytes (M), and lymphocytes (L) as follows: SIRI = N × M/L.

A total of 3,964 elderly patients with HF from the MIMIC-III database and 261 patients from the Second Affiliated Hospital of Wenzhou Medical University from May 1, 2020, to June 30, 2021 were included in this study. Figure 1 showed the flowchart of study cohort selection from the MIMIC-III database. The study population from MIMIC-III database were stratified respectively on SIRI tertiles and 90-day all-cause mortality, and the baseline characteristics of the subjects are displayed in Table 1, Supplementary Table S1 (see Additional file 1). As shown in Table 1, Supplementary Table S1, patients with high SIRI values were more likely to be older with a higher heart rate, respiratory rate, serum creatinine, platelet count, WBC count, RDW, BUN, anion gap, SOFA score, and SAPS II score. However, they had a lower SBP, DBP and MAP. Furthermore, these patients tended to have a history of atrial fibrillation, COPD, respiratory failure, SIRS, and peripheral vascular disease. Patients with high SIRI values were more likely to require RRT. Likewise, the patients from the Second Affiliated Hospital of Wenzhou Medical University were equally divided into three groups according to the SIRI value, and the comparison of their baseline characteristics was summarized in Supplementary Table S2 (see Additional file 2). It was worth noting that we observed similar baseline features. Patients with high SIRI values were more likely to be older with higher heart rate, respiratory rate, platelet counts, alanine aminotransferase (ALT), WBC counts, NT-proBNP, cTN-I and CRP values. They had a lower SBP, DBP, LVEF. Moreover, these patients tended to have a history of coronary atherosclerotic heart disease, valvular disease, chronic kidney disease, and atherosclerosis.

**Table 1:** Baseline characteristics of the study population.

**Characteristics**	**SIRI**			***P* value**
	**<2.0**	**2.0–4.9**	**>4.9**	
Number of patients	1,321	1,321	1,322	
Age, years	77.51 ± 9.45	77.74 ± 9.34	77.94 ± 9.02	0.496
**Sex**, ***n*** **(%)**				0.007
Female	714 (54.05)	672 (50.87)	634 (47.96)	
Male	607 (45.95)	649 (49.13)	688 (52.04)	
**Ethnicity**, ***n*** **(%)**				<0.001
Black	259 (19.61)	145 (10.98)	84 (6.35)	
White	917 (69.42)	1038 (78.58)	1092 (82.60)	
Other	145 (10.98)	138 (10.45)	146 (11.04)	
**Vital signs**				
Heart rate, beats/min	80.80 ± 15.34	83.32 ± 15.51	85.99 ± 15.80	<0.001
SBP, mmHg	117.40 ± 17.78	116.46 ± 16.79	114.07 ± 16.37	<0.001
DBP, mmHg	57.90 ± 10.28	57.52 ± 9.86	56.36 ± 9.81	<0.001
MAP, mmHg	74.35 ± 10.70	73.85 ± 10.35	72.37 ± 9.81	<0.001
Respiratory rate, times/min	19.48 ± 3.64	19.96 ± 3.83	20.71 ± 4.01	<0.001
Temperature, °C	36.61 ± 0.57	36.66 ± 0.59	36.69 ± 0.63	0.002
SpO2, %	96.88 ± 2.09	96.67 ± 2.30	96.68 ± 2.11	0.021
**Laboratory parameters**				
SIRI, 10^9^/L	1.17 ± 0.52	3.32 ± 0.83	14.67 ± 20.31	<0.001
Neutrophil count, 10^9^/L	5.61 ± 2.66	8.93 ± 3.20	14.46 ± 6.73	<0.001
Monocyte count, 10^9^/L	0.33 ± 0.28	0.45 ± 0.22	0.78 ± 0.72	<0.001
Lymphocyte count, 10^9^/L	2.24 ± 16.38	1.24 ± 0.75	0.96 ± 0.64	0.001
White blood cell count, 10^9^/L	8.50 ± 17.32	10.92 ± 3.80	16.81 ± 7.90	<0.001
Hemoglobin, g/dl	10.82 ± 2.24	10.89 ± 2.20	11.04 ± 2.16	0.028
Platelet count, 10^9^/L	227.56 ± 105.66	257.05 ± 112.50	273.73 ± 129.86	<0.001
RDW, %	15.84 ± 2.18	15.84 ± 2.15	15.89 ± 2.22	0.740
Glucose, mg/dl	141.26 ± 48.06	146.69 ± 49.30	147.75 ± 49.52	0.001
Serum creatinine, mg/dl	1.91 ± 1.55	1.97 ± 1.67	2.00 ± 1.48	0.324
Blood urea nitrogen, mg/dl	38.12 ± 26.28	40.37 ± 26.78	42.80 ± 28.50	<0.001
Anion gap, mg/dl	15.43 ± 4.04	16.01 ± 4.19	16.76 ± 4.27	<0.001
**Comorbidities**, ***n*** **(%)**	671 (50.79)	692 (52.38)	760 (57.49)	0.002
**Atrial fibrillation**				
CAD	600 (45.42)	598 (45.27)	596 (45.08)	0.985
Valvular disease	391 (29.60)	405 (30.66)	387 (29.27)	0.718
CKD^a^	504 (38.15)	509 (38.53)	503 (38.05)	0.965
Liver disease^b^	48 (3.63)	44 (3.33)	43 (3.25)	0.850
COPD	85 (6.43)	106 (8.02)	109 (8.25)	0.158
Respiratory failure	457 (34.60)	518 (39.21)	613 (46.37)	<0.001
Pneumonia	327 (24.75)	450 (34.07)	564 (42.66)	<0.001
Hypertension	976 (73.88)	941 (71.23)	907 (68.61)	0.011
Peripheral vascular disease	196 (14.84)	198 (14.99)	203 (15.36)	0.929
Complicated diabetes	159 (12.04)	187 (14.16)	162 (12.25)	0.200
SIRS	1315 (99.55)	1316 (99.62)	1321 (99.92)	0.173
**Scoring systems**				
SOFA	4.69 ± 2.84	4.74 ± 2.79	5.24 ± 3.06	<0.001
SAPSII	41.08 ± 11.26	41.74 ± 11.72	44.26 ± 12.11	<0.001
Clinical outcomes, *n* (%)				
90-day all-cause mortality	212 (16.05)	284 (21.50)	353 (26.70)	<0.001
30-day all-cause mortality	128 (9.69)	172 (13.02)	223 (16.87)	<0.001
One-year all-cause mortality	373 (28.24)	419 (31.72)	498 (37.67)	<0.001
Length of hospital stay, day	9.62 ± 8.46	10.19 ± 8.75	11.59 ± 9.31	<0.001
Length of ICU stay, day	4.14 ± 4.69	4.64 ± 5.59	5.62 ± 6.83	<0.001
Need for RRT	118 (8.93)	133 (10.07)	149 (11.27)	0.137

*SIRI, systemic inflammation response index. SIRI is calculated using the counts of peripheral venous blood neutrophils (N), monocytes (M), and lymphocytes (L) as follows: SIRI = N*

^*^ M/L. SBP, systolic blood pressure; DBP, diastolic blood pressure; MAP, mean arterial pressure; SpO2, pulse oximetry-derived oxygen saturation; RDW, red cell volume distribution width; SIRS, system inflammatory response syndrome; CKD, chronic kidney disease; COPD, chronic obstructive pulmonary disease; CAD, coronary artery disease; SOFA, sequential organ failure assessment; SAPS II, simplified acute physiology score II. RRT, renal replacement therapy; ICU, Intensive Care Unit. Data were presented as the mean ± SD and n (%).

a *CKD contains CKD stage I–V and end stage renal disease.*

b *Liver disease refers to the pathological changes that occur in the liver, including viral hepatitis, cirrhosis, fatty liver, alcoholic liver disease, portal hypertension, hepatic encephalopathy, hepatorenal syndrome, liver necrosis, and many other liver diseases and their complications. Excluding liver cancer*.

### Relationship Between the SIRI and the Clinical Outcomes

**Figure 2:**
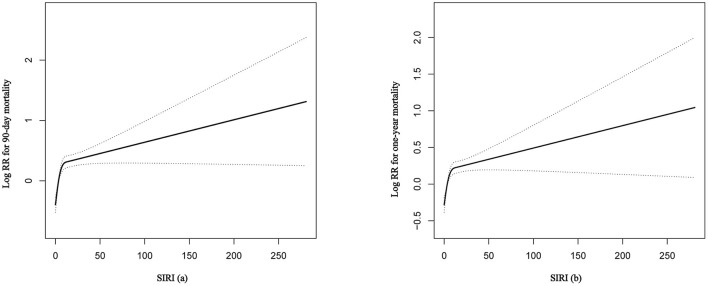
The smooth curve fitting demonstrates the relationship between SIRI and the 90-day and 1-year all-cause mortality. The resulting figures show the predicted log (relative risk) in the *y*-axis and the continuous variables in the *x*-axis. RR, relative risk; SIRI, systemic inflammation response index.

Firstly, the smooth curve fitting intuitively displayed that the 90-day and 1-year all-cause mortality increased significantly with an increase in SIRI (Figures 2A,B). This analysis was performed using both logarithmic transformed and untransformed data. Log relative risk (Log RR) was converted to a relative risk using antilog. Secondly, the clinical outcomes of the subjects were displayed in Table 2 across the tertiles of baseline SIRI. In Table 2 the study population was from MIMIC-III database. A high SIRI was associated with an increased risk of 90-day and 1-year all-cause mortality in elderly patients with HF. In model 2, a higher SIRI was associated with an increased risk of all-cause mortality after adjusting for age, gender, and ethnicity compared with the first tertile. In model 3, after adjusting for more confounding factors, SIRI was shown to be an independent predictor of 90-day and 1-year all-cause mortality in critically ill elderly patients with HF (tertile 3 vs. tertile 1: adjusted HR, 95% CI: 1.41 (1.18, 1.68), 1.19 (1.03, 1.37); *p* trend = 0.0013, 0.0260; respectively). In addition, as shown in Table 2, elevated SIRI was associated with increased the length of hospital or ICU stay after adjusting for multiple confounders (tertile 3 vs. tertile 1: *β*, 95% CI: 0.85 (0.16, 1.54); 0.62 (0.18, 1.06); *p* trend = 0.0095, 0.0046; respectively). After adjusting for multiple confounders, the logistic regression model reveled that the patients with higher SIRI levels were more likely to require RRT (tertile 3 vs. tertile 1: OR, 95% CI: 1.55 (1.06, 2.28); *p* trend = 0.0459). A similar trend in these outcomes was observed in the SIRI group inclusion according to quintiles (Supplementary Table S3, see Additional file 3). Finally, subgroup analysis of the subjects from the MIMIC-III database showed that there were no significant interactions between the variables (Table 3).

**Table 2:** Association between SIRI tertiles and clinical outcomes of in critically ill patients with congestive heart failure.

**Clinical outcomes**	**Model 1**		**Model 2**		**Model 3**	
	**HR (95% CI)**	***P* value**	**HR (95% CI)**	***P* value**	**HR (95% CI)**	***P* value**
Primary outcome						
90-day all-cause mortality^a^						
<2.0	1.0		1.0		1.0	
2.0–4.9	1.39 (1.16, 1.66)	0.0003	1.37 (1.15, 1.64)	0.0005	1.31 (1.09, 1.57)	0.0038
>4.9	1.77 (1.49, 2.10)	<0.0001	1.72 (1.45, 2.05)	<0.0001	1.41 (1.18, 1.68)	0.0002
*P for trend*		<0.0001		<0.0001		0.0013
Secondary outcomes						
One-year all-cause mortality^a^						
<2.0	1.0		1.0		1.0	
2.0–4.9	1.17 (1.02, 1.35)	0.0246	1.16 (1.01, 1.33)	0.0387	1.10 (0.95, 1.27)	0.1890
>4.9	1.46 (1.28, 1.67)	<0.0001	1.42 (1.24, 1.63)	<0.0001	1.19 (1.03, 1.37)	0.0188
*P for trend*		<0.0001		<0.0001		0.0260
Length of hospital stay^b^						
<2.0	0		0		0	
2.0–4.9	0.57 (−0.11, 1.24)	0.0990	0.46 (−0.21, 1.14)	0.1775	0.13 (−0.52, 0.78)	0.6869
>4.9	1.97 (1.30, 2.64)	<0.0001	1.81 (1.13, 2.49)	<0.0001	0.85 (0.16, 1.54)	0.0158
*P for trend*		<0.0001		<0.0001		0.0095
Length of ICU stay^b^						
<2.0	0		0		0	
2.0–4.9	0.50 (0.06, 0.94)	0.0256	0.47 (0.03, 0.91)	0.0378	0.20 (−0.22, 0.61)	0.3492
>4.9	1.49 (1.05, 1.93)	<0.0001	1.43 (0.98, 1.87)	<0.0001	0.62 (0.18, 1.06)	0.0055
*P for trend*		<0.0001		<0.0001		0.0046
Renal replacement therapy^c^						
<2.0	1.0		1.0		1.0	
2.0–4.9	1.14 (0.88, 1.48)	0.3199	1.29 (0.99, 1.69)	0.0630	1.38 (0.94, 2.00)	0.0974
>4.9	1.30 (1.00, 1.67)	0.0466	1.60 (1.22, 2.09)	0.0006	1.55 (1.06, 2.28)	0.0248
*P for trend*		0.0536		0.0010		0.0459

a *: Cox proportional hazards regression models were used to calculate hazard ratios (HR) with 95% confidence intervals (CI).*

b *: Linear regression model were used to calculate β value with 95% confidence intervals (CI).*

c *: Logistic regression models were used to calculate odds ratios (OR) with 95% confidence intervals (CI).*

*Model 1 covariates were adjusted for nothing; Model 2 covariates were adjusted for age, sex and ethnicity; Model 3: a: covariates were adjusted for age, sex, ethnicity, systolic blood pressure, diastolic blood pressure, system inflammatory response syndrome, serum creatinine, hemoglobin, white blood cell count, platelet count, red cell volume distribution width, atrial fibrillation, coronary artery disease, chronic kidney disease, respiratory failure, pneumonia, hypertension; b: covariates were adjusted for age, sex, ethnicity, blood urea nitrogen, white blood cell count, platelet count, atrial fibrillation, respiratory failure, pneumonia, SOFA, SASP II; c: covariates were adjusted for age, sex, ethnicity, diastolic blood pressure, respiratory rate, anion gap, serum creatinine, white blood cell count, platelet count, red cell volume distribution width, atrial fibrillation, chronic kidney disease, respiratory failure, pneumonia, SOFA, SASP II*.

**Table 3:** Subgroup analysis of the associations between 90-day all-cause mortality and the SIRI.

	**No. of patients**	**SIRI**			***P* for interaction**
		**<2.0**	**2.0–4.9**	**>4.9**	
**Age, years**					0.7111
<78.4	1,982	1.0	1.38 (1.05, 1.83)	1.79 (1.37, 2.34)	
≥78.4	1,982	1.0	1.39 (1.10, 1.75)	1.77 (1.41, 2.20)	
**Sex**					0.2708
Male	2,020	1.0	1.26 (0.97, 1.63)	1.84 (1.44, 2.34)	
Female	1,944	1.0	1.50 (1.17, 1.92)	1.69 (1.33, 2.15)	
**Atrial fibrillation**					0.1334
No	1,841	1.0	1.60 (1.21, 2.11)	2.14 (1.64, 2.80)	
Yes	2,123	1.0	1.24 (0.98, 1.57)	1.51 (1.21, 1.88)	
**CAD**					0.0170
No	2,170	1.0	1.25 (1.00, 1.56)	1.45 (1.17, 1.80)	
Yes	1,794	1.0	1.66 (1.23, 2.23)	2.41 (1.82, 3.19)	
**Valvular disease**					0.5037
No	2,781	1.0	1.30 (1.04, 1.61)	1.66 (1.35, 2.04)	
Yes	1,183	1.0	1.58 (1.16, 2.15)	2.04 (1.51, 2.75)	
**Hypertension**					0.8387
No	1,140	1.0	1.41 (1.04, 1.92)	1.68 (1.25, 2.26)	
Yes	2,824	1.0	1.36 (1.09, 1.69)	1.78 (1.45, 2.20)	
**Complicated diabetes**					0.7627
No	3,456	1.0	1.39 (1.15, 1.68)	1.74 (1.45, 2.08)	
Yes	508	1.0	1.40 (0.83, 2.36)	2.04 (1.23, 3.37)	
**Peripheral vascular disease**					0.3251
No	3,367	1.0	1.47 (1.21, 1.78)	1.81 (1.51, 2.19)	
Yes	597	1.0	1.01 (0.64, 1.60)	1.56 (1.03, 2.38)	
**Respiratory failure**					0.6203
No	2,376	1.0	1.38 (1.08, 1.77)	1.81 (1.43, 2.31)	
Yes	1,588	1.0	1.34 (1.03, 1.73)	1.55 (1.21, 1.97)	
**COPD**					0.5458
No	3,664	1.0	1.40 (1.16, 1.69)	1.82 (1.52, 2.17)	
Yes	300	1.0	1.21 (0.64, 2.27)	1.29 (0.69, 2.40)	
**CKD**					0.3040
No	2,448	1.0	1.48 (1.18, 1.85)	1.72 (1.38, 2.14)	
Yes	1,516	1.0	1.25 (0.93, 1.67)	1.86 (1.42, 2.44)	
**MAP, mmHg**					0.5808
<72.1	1,979	1.0	1.32 (1.04, 1.68)	1.57 (1.25, 1.97)	
≥72.1	1,979	1.0	1.47 (1.12, 1.92)	2.00 (1.54, 2.60)	
**Hemoglobin, g/dl**					0.2972
<10.8	1,917	1.0	1.33 (1.05, 1.68)	1.49 (1.18, 1.88)	
≥10.8	2,046	1.0	1.48 (1.13, 1.94)	2.16 (1.67, 2.78)	
**RDW, %**					0.1289
NA	1,915	1.0	1.70 (1.26, 2.29)	2.28 (1.71, 3.02)	
NA	2,046	1.0	1.23 (0.98, 1.53)	1.52 (1.22, 1.88)	
**White blood cell count, 10^9^/L**					0.4807
<10.6	1,979	1.0	1.35 (1.08, 1.69)	1.73 (1.26, 2.38)	
≥10.6	1,985	1.0	1.24 (0.89, 1.74)	1.49 (1.08, 2.04)	
**Platelet count, 10** ^**9**^ **/L**					0.1004
<232.0	1,964	1.0	1.21 (0.96, 1.54)	1.70 (1.35, 2.13)	
≥232.0	1,999	1.0	1.73 (1.31, 2.29)	2.05 (1.57, 2.69)	
**Blood urea nitrogen, mg/dl**					0.4992
<32	1,957	1.0	1.43 (1.08, 1.89)	1.77 (1.34, 2.32)	
≥32	2,003	1.0	1.29 (1.02, 1.62)	1.63 (1.31, 2.02)	
**Serum creatinine, mg/dl**					0.1919
<1.4	1,803	1.0	1.45 (1.10, 1.91)	1.94 (1.48, 2.53)	
≥1.4	2,156	1.0	1.34 (1.06, 1.69)	1.63 (1.31, 2.03)	
**Anion gap, mg/dl**					0.0581
<16	1,905	1.0	1.49 (1.15, 1.92)	2.02 (1.57, 2.60)	
≥16	2,027	1.0	1.24 (0.97, 1.59)	1.51 (1.20, 1.91)	
**SAPSII score**					0.3249
<41	1,978	1.0	1.39 (1.03, 1.88)	1.93 (1.44, 2.59)	
≥41	1,986	1.0	1.35 (1.08, 1.68)	1.48 (1.20, 1.83)	
**SOFA score**					0.2252
<4	1,474	1.0	1.38 (0.98, 1.95)	2.18 (1.57, 3.03)	
≥4	2,490	1.0	1.39 (1.13, 1.71)	1.57 (1.29, 1.92)	

*SIRI, systemic inflammation response index; MAP, mean arterial pressure; RDW, red cell volume distribution width; CKD, chronic kidney disease; COPD, chronic obstructive pulmonary disease; CAD, coronary artery disease; SOFA, sequential organ failure assessment; SAPS II, simplified acute physiology score II. HR (95% CI) were derived from Cox proportional hazards regression models. Covariates were adjusted as in model 1 (Table 2)*.

### Sensitivity and Specificity of SIRI

**Figure 3:**
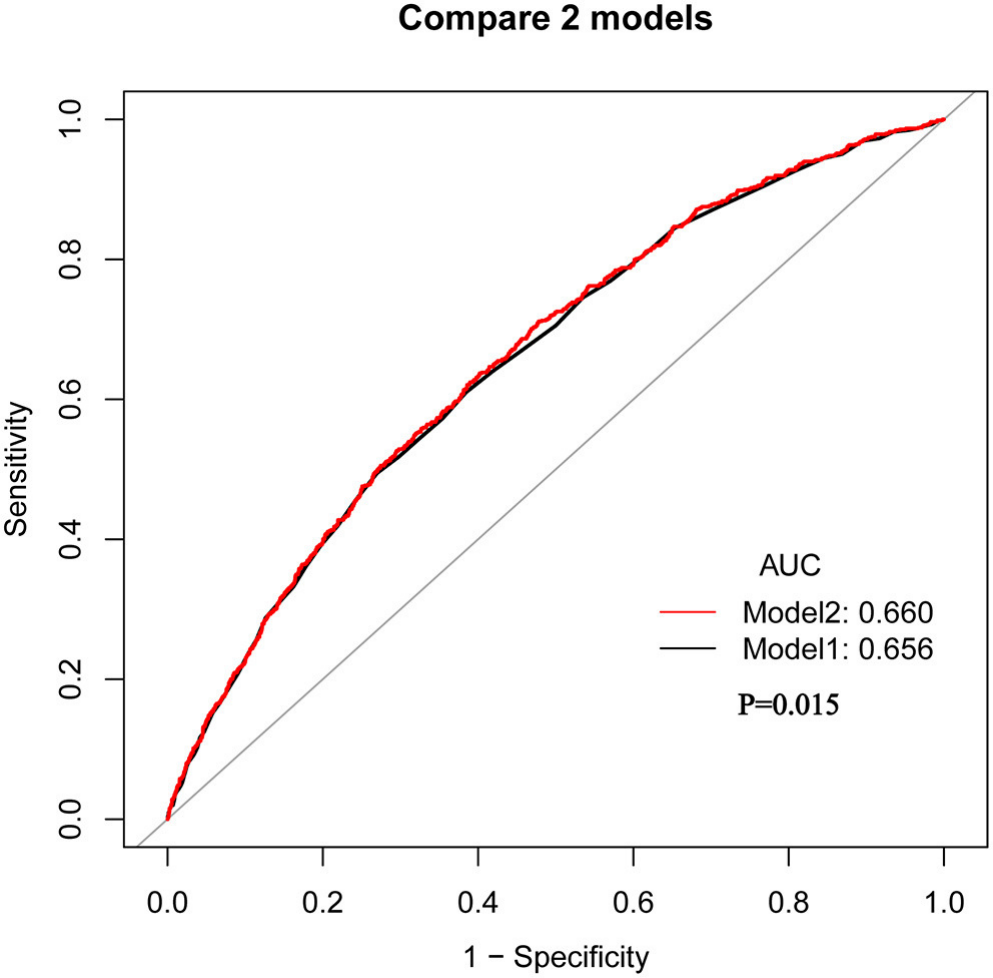
The ROC analysis shows the predictive value of SIRI for 90-day all-cause mortality in HF patients. Model 1: Parameter only include SAPSII; Model 2: Parameters include SAPSII and SIRI.

ROC curve analysis was performed to assess the potential prognostic value of SIRI for 90-day all-cause mortality in HF patients (Figure 3). We conducted two predictive models, and the model 1 parameter was SAPSII, and the parameters of the model 2 included SIRI and SAPSII. We found that the area under the curves (AUCs) for model 1 and model 2 were 0.656 (95% CI: 0.635–0.676) and 0.660 (95% CI: 0.640–0.681), respectively. Comparing AUCs, model 2 was a better predictive model than SAPSII alone (*P* = 0.015), and SIRI significantly enhanced the prediction efficiency of SAPSII. ROC curve analysis suggested that the SIRI had good sensitivity and specificity for the risk assessment of death in patients with HF.

### Association Between CRP and SIRI

**Figure 4:**
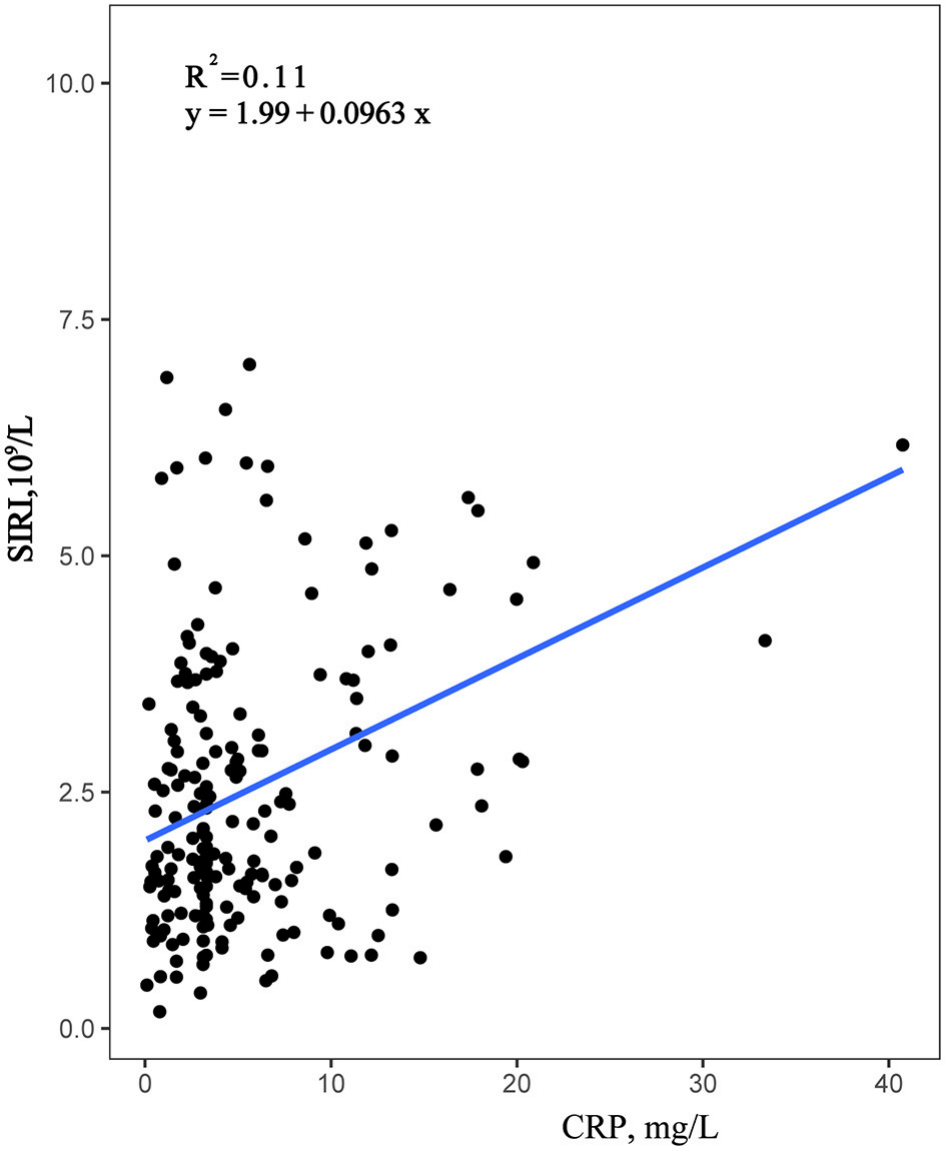
Simple linear regression analysis between SIRI and CRP. SIRI was associated with CRP.

Data were collected from the Second Affiliated Hospital of Wenzhou Medical University, using Pearson correlation analysis, confirmed that CRP was significantly positively correlated with neutrophils and SIRI, with correlation coefficients of 0.343 and 0.259, respectively, both *P* < 0.001 (Table 4). Moreover, the correlation between SIRI and CRP was higher than the correlation between CRP and Neutrophils. However, the correlation between CRP and monocytes and lymphocytes was not statistically significant. Furthermore, a simple linear regression analysis was performed with SIRI on the *Y*-axis and CRP on the *X*-axis (Figure 4). The results indicated that SIRI was significantly associated with CRP. Therefore, SIRI can be used as an additional complementary inflammatory marker to predict risk in HF.

**Table 4:** Correlation analysis between CRP and SIRI, N, M, and L.

	**CRP, mg/L**	**SIRI, 10^9^/L**	**N, 10^9^/L**	**L, 10^9^/L**	**M, 10^9^/L**
CRP, mg/L	1				
SIRI, 10^9^/L	0.343^**^	1			
N, 10^9^/L	0.259^**^	0.593^**^	1		
L, 10^9^/L	−0.120	−0.470^**^	0.323^**^	1	
M, 10^9^/L	0.018	−0.089	−0.118	-0.104	1

** *At 0.01 level (two tailed), the correlation was significant.*

*SIRI, systemic inflammation response index; N, neutrophils count; M, monocytes count; L, lymphocytes count; CRP, C reactive protein*.

## Discussion

This study was focused on the association of SIRI with adverse prognosis in elderly patients with HF. Cox regression model after adjusting for multiple variables and the smooth curve fitting both showed that a high SIRI was significantly associated with all-cause mortality in elderly patients with HF. However, other factors might also affect mortality. As such, we conducted a subgroup analysis based on some clinical parameters and comorbidities, and found that there was no significant interaction, which enhanced the reliability of our findings. In addition, we found that length of stay or ICU stay in patients with heart failure significantly increased with increased SIRI. Nevertheless, this study did not elucidate the precise mechanisms underlying the association between SIRI and these outcomes.

HF is a chronic disease with high mortality rates. The estimated 1-year mortality rate is more than 20% (18). The most powerful predictors of mortality are older age (more than 60), the presence of DM, and a lower LVEF (19). In this study, the subjects were elderly patients with HF over 60 years old. Because of poor prognosis in elderly HF, it's urgent to develop an efficient biomarker for quick identification of high-risk patients of HF. So that clinicians can give relevant treatment in time and improve the prognosis.

Chronic inflammation is observed to be more common in chronic diseases, such as cancers, DM, and chronic kidney disease (20–22). It has been confirmed that the SIRI can be used for the prognostication of patients with various malignant tumors. Systemic inflammation is considered to be an important cause of myocardial disorder and end-stage HF, which plays a pivotal role in the development and progression of HF (23). Myocardial inflammation is sustained in individuals with ischaemic or non-ischaemic HF and involves a variety of inflammatory cells (including neutrophils, monocytes, macrophages and lymphocytes) and cytokines (such as IL-6, TNF, etc.) (24). So, SIRI could better reflect the state of long-term inflammation of HF. Neutrophils play a crucial role in the innate immune system coordinating inflammation-resolution and host defense mechanisms (25). Circulating monocytes infiltrate the heart early after myocardial injury and participate in inflammation and repair processes, thereby affecting left ventricular remodeling (26). Lymphocytes, a marker of the regulatory pathway, are associated with adaptive long-term immune response (27, 28). Immune response stimulates lymphocyte apoptosis, resulting in lymphocytopenia (29, 30). Lymphopenia has been demonstrated to be an independent prognostic factor of HF mortality (31). However, a single indicator of inflammation is not sufficient to predict the severity of inflammation. SIRI is a compound inflammatory index based on neutrophils, monocytes and lymphocytes. It is less affected by the absolute number of a single index and has predictive ability in a large range. Moreover, the combination of specific and non-specific immune pathways has an important role in the outcome. Among them, neutrophils, monocytes and their cytokines are mainly related to non-specific pathways, and circulating lymphocytes in the blood are considered to be mainly related to specific pathways. Compared with a single index, the significance of inflammation is more significant and stable. The SIRI, which combines three inflammatory biomarkers, is a comprehensive, easily accessible, and inexpensive indicator of chronic low-grade inflammation. SIRI also includes the NLR and MLR, which might be a more sensitive and useful inflammatory biomarkers than any of these alone. It is worth noting that SIRI, as the combination of NLR and MLR, may have similar scenarios of treatment. Nebivolol has antioxidant and anti-inflammatory properties and could decrease the NLR (32). Amlodipine and valsartan could decrease the NLR in patients with newly diagnosed hypertension (33). Atorvastatin could significantly decrease the NLR in patients with hypercholesterolemia (34). Colchicine, which can inhibit the inflammatory response mediated by monocytes and neutrophils, could reduce major adverse cardiovascular events (MACE) in patients with CAD (35, 36). Accordingly, SIRI has a great potential to predict the prognosis of elderly patients with HF and add clinical relevance to guide HF management.

Moreover, the ROC curve suggested that the combination of SIRI and SAPS II could better predict the mortality rate of elderly HF patients. On the one hand, it suggested that inflammatory process plays a very important role in the development of heart failure. On the other hand, it also provided an important support for the inclusion of inflammatory markers in the establishment of new models.

In this study, we also found that patients with high SIRI were more likely to require RRT. Rapid deterioration of cardiac function resulting in acute kidney injury (AKI) is commonly defined as a cardiorenal syndrome (CRS) and is associated with high mortality. Older patients may be more prone to CRS because of complications such as hypertension or diabetes, etc. The BEST Kidney study that enrolled a large cohort of critically ill patients with AKI demonstrated that older age was independently associated with high mortality (37). The mortality rate in AKI patients requiring RRT was more than 80% (38). Therefore, the severity of these patients has a high degree of disease. In the present study, patients with a high SIRI were more likely to require RRT, which suggested that SIRI was associated with the severity of disease.

As a reflection of inflammation, CRP is the most widely clinically applied inflammatory marker (39). However, CRP usually reflects an acute inflammatory process and does not fully reflect the long-term persistence of low-grade inflammation in HF. Pearson correlation analysis results showed that a statistically significant positive correlation between SIRI and CRP, which indicated SIRI might be a useful complementary inflammatory marker and highlighted the importance of systemic inflammation as a determinant of outcome in patients with HF. Moreover, SIRI could better reflect the long-term inflammatory change state of heart failure than a single CRP.

To the best of our knowledge, no other studies have evaluated the association between SIRI and clinical outcomes in elderly patients with HF. Moreover, we used a large cohort of patients, which increases the reliability of our findings. However, several limitations of this study should also be noted. First, this was a single-center retrospective study, which may affect the generalization of the results. Therefore, a further study enrolling patients from multiple centers should be carried out. Second, SIRI is a dynamic biomarker, and thus, the study findings may be affected by bias resulting from the use of the first blood sample. Third, there were too many missing important variables in the MIMIC-III data base, which may make the model imperfect. Fourth, the results obtained in this study were not fully verified by clinical data, because we did not further follow up the survival of patients after discharge. Despite the shortcomings, SIRI shows a good prognostic ability in elderly patients with HF.

## Conclusions

SIRI could be a novel promising inflammatory biomarker for predicting all-cause mortality in elderly patients with HF. And the patients with higher SIRI values had the longer length of hospital or ICU stay and were more likely to require for RRT. Of note, this study also verified a statistically significant positive correlation between SIRI and the inflammatory marker CRP, highlighting the importance of systemic inflammation as a determinant of outcome in patients with HF. However, further prospective cohort studies are required to validate these study findings.

## Data Availability Statement

The original contributions presented in the study are included in the article/Supplementary Material, further inquiries can be directed to the corresponding author/s.

## Author Contributions

All authors listed have made a substantial, direct, and intellectual contribution to the work and approved it for publication.

## Conflict of Interest

The authors declare that the research was conducted in the absence of any commercial or financial relationships that could be construed as a potential conflict of interest.

## Publisher's Note

All claims expressed in this article are solely those of the authors and do not necessarily represent those of their affiliated organizations, or those of the publisher, the editors and the reviewers. Any product that may be evaluated in this article, or claim that may be made by its manufacturer, is not guaranteed or endorsed by the publisher.
