# Supplementary material for: Systemic Inflammation Response Index Is a Promising Prognostic Marker in Elderly Patients With Heart Failure: A Retrospective Cohort Study
Source: Front Cardiovasc Med. 2022 Jul 14;9:871031. doi: 10.3389/fcvm.2022.871031 (PMC9330028; doi:10.3389/fcvm.2022.871031)
Supplement: Supplementary file 1 [file Table_1.docx]

TableS1. Baseline characteristics of 90-day all-cause death in critical ill patients with heart failure.

| **Clinical parameters** | Survival within 90-day | Death within 90-day | *P value* |
| --- | --- | --- | --- |
| **Number of patients** | 3115 | 849 |  |
| Age, years | 77.12 ± 9.35 | 79.98 ± 8.59 | <0.001 |
| Sex, n (%) |  |  | 0.009 |
| Female | 1621 (52.04) | 399 (47.00) |  |
| Male | 1494 (47.96) | 450 (53.00) |  |
| Ethnicity, n (%) |  |  | 0.005 |
| Black | 411 (13.19) | 77 (9.07) |  |
| White | 2372 (76.15) | 675 (79.51) |  |
| Other | 332 (10.66) | 97 (11.43) |  |
| **Vital signs** |  |  |  |
| Heart rate, beats/minute | 82.70 ± 15.48 | 85.83 ± 16.22 | <0.001 |
| SBP, mmHg | 116.90 ± 17.00 | 112.56 ± 16.78 | <0.001 |
| DBP, mmHg | 57.65 ± 10.09 | 55.82 ± 9.58 | <0.001 |
| MAP, mmHg | 73.90 ± 10.35 | 72.12 ± 10.09 | <0.001 |
| Respiratory rate, times/minute | 19.91 ± 3.76 | 20.57 ± 4.16 | <0.001 |
| Temperature, ℃ | 36.67 ± 0.58 | 36.59 ± 0.64 | <0.001 |
| SpO2, % | 96.74 ± 2.10 | 96.77 ± 2.40 | 0.698 |
| **Laboratory parameters** |  |  |  |
| SIRI, 10^9^/L | 5.84 ± 11.73 | 8.39 ± 17.25 | <0.001 |
| Neutrophil count, 10^9^/L | 9.40 ± 5.68 | 10.67 ± 6.32 | <0.001 |
| Monocyte count, 10^9^/L | 0.51 ± 0.46 | 0.57 ± 0.62 | 0.002 |
| Lymphocyte count, 10^9^/L | 1.37 ± 2.87 | 1.89 ± 19.76 | 0.159 |
| White blood cell count, 10^9^/L | 11.66 ± 7.07 | 13.62 ± 21.38 | <0.001 |
| Hemoglobin, g/dL | 10.97 ± 2.23 | 10.73 ± 2.07 | 0.004 |
| Platelet count, 10^9^/L | 252.47 ± 116.10 | 253.99 ± 124.75 | 0.739 |
| RDW, % | 15.73 ± 2.11 | 16.34 ± 2.36 | <0.001 |
| Glucose, mg/dL | 145.05 ± 49.03 | 145.91 ± 49.07 | 0.652 |
| Serum creatinine, mg/dl | 1.94 ± 1.60 | 2.04 ± 1.46 | 0.105 |
| Blood urea nitrogen, mg/dl | 38.80 ± 26.29 | 46.41 ± 29.82 | <0.001 |
| Anion gap, mg/dl | 15.98 ± 4.11 | 16.40 ± 4.52 | 0.010 |
| **Comorbidities, n (%)** |  |  |  |
| Atrial fibrillation | 1626 (52.20) | 497 (58.54) | 0.001 |
| CAD | 1455 (46.71) | 339 (39.93) | <0.001 |
| Valvular disease | 892 (28.64) | 291 (34.28) | 0.001 |
| CKD ^#^ | 1191 (38.23) | 325 (38.28) | 0.981 |
| Liver disease ^$^ | 94 (3.02) | 41 (4.83) | 0.010 |
| COPD | 234 (7.51) | 66 (7.77) | 0.798 |
| Respiratory failure | 1153 (37.01) | 435 (51.24) | <0.001 |
| Pneumonia | 950 (30.50) | 391 (46.05) | <0.001 |
| Hypertension | 2268 (72.81) | 556 (65.49) | <0.001 |
| Peripheral vascular disease | 469 (15.06) | 128 (15.08) | 0.988 |
| Complicated diabetes | 403 (12.94) | 105 (12.37) | 0.660 |
| SIRS | 3107 (99.74) | 845 (99.53) | 0.314 |
| RRT | 299 (9.60) | 101 (11.90) | 0.049 |
| **Scoring systems** |  |  |  |
| SAPSII | 40.97 ± 11.12 | 47.47 ± 12.68 | <0.001 |
| SOFA | 4.64 ± 2.77 | 5.81 ± 3.22 | <0.001 |
| **Length of ICU stay, day** | 4.51 ± 5.58 | 5.88 ± 6.44 | <0.001 |
| **Length of hospital stay, day** | 10.18 ± 8.59 | 11.50 ± 9.82 | <0.001 |

**Abbreviations:** SIRI: systemic inflammation response index. SIRI is calculated using the counts of peripheral venous blood neutrophils (N), monocytes (M), and lymphocytes (L) as follows: SIRI=N*M/L; SBP: systolic blood pressure; DBP: diastolic blood pressure; MAP: mean arterial pressure; SpO2: pulse oximetry-derived oxygen saturation; RDW: red cell volume distribution width; SIRS: system inflammatory response syndrome; CKD: chronic kidney disease; COPD: chronic obstructive pulmonary disease ; CAD: coronary artery disease; SOFA: sequential organ failure assessment; SAPS II: simplified acute physiology score II. RRT: renal replacement therapy; ICU: Intensive Care Unit. Data were presented as the mean ± SD and n (%).

#: CKD contains CKD stage I-V and end stage renal disease. $: Liver disease refers to the pathological changes that occur in the liver, including viral hepatitis, cirrhosis, fatty liver, alcoholic liver disease, portal hypertension, hepatic encephalopathy, hepatorenal syndrome, liver necrosis, and many other liver diseases and their complications. Excluding liver cancer.
