# Supplementary material for: Systemic Inflammation Response Index Is a Promising Prognostic Marker in Elderly Patients With Heart Failure: A Retrospective Cohort Study
Source: Front Cardiovasc Med. 2022 Jul 14;9:871031. doi: 10.3389/fcvm.2022.871031 (PMC9330028; doi:10.3389/fcvm.2022.871031)
Supplement: Supplementary file 2 [file Table_2.docx]

Table S2. Baseline characteristics of the study population.

| **Characteristics** |  | SIRI |  | *P value* |
| --- | --- | --- | --- | --- |
|  | <1.84 | 1.84-3.81 | >3.81 |  |
| Number of patients | 87 | 87 | 87 |  |
| Age, years | 74.01 ± 10.62 | 75.07 ± 12.64 | 76.74 ± 9.92 | 0.267 |
| Sex, n (%) |  |  |  | 0.827 |
| Female | 36 (41.38) | 38 (43.68) | 34 (39.08) |  |
| Male | 51 (58.62) | 49 (56.32) | 53 (60.92) |  |
| **Vital signs** |  |  |  |  |
| Temperature, ℃ | 36.77 ± 0.49 | 36.75 ± 0.51 | 36.89 ± 0.49 | 0.117 |
| Respiratory rate, times/minute | 19.55 ± 2.52 | 20.14 ± 6.40 | 20.56 ± 3.34 | 0.318 |
| Heart rate, beats/minute | 83.85 ± 19.88 | 84.70 ± 17.92 | 87.77 ± 19.64 | 0.367 |
| SBP, mmHg | 140.33 ± 22.12 | 140.47 ± 22.95 | 134.55 ± 27.99 | 0.193 |
| DBP, mmHg | 77.10 ± 15.49 | 78.29 ± 14.79 | 74.52 ± 14.43 | 0.235 |
| SpO2, ％ | 97.19 ± 2.16 | 97.32 ± 2.24 | 95.87 ± 5.32 | 0.021 |
| **Laboratory parameters** |  |  |  |  |
| CRP，mg/L | 4.98 ± 5.26 | 16.11 ± 33.77 | 33.86 ± 42.18 | <0.001 |
| SIRI，10^9^/L | 1.26 ± 0.41 | 2.76 ± 0.57 | 8.44 ± 6.60 | <0.001 |
| White blood cell count, 10^9^/L | 7.02 ± 10.46 | 7.32 ± 2.79 | 9.55 ± 4.45 | 0.027 |
| Neutrophil count, 10^9^/L | 3.57 ± 1.25 | 5.27 ± 2.13 | 8.02 ± 4.09 | <0.001 |
| Lymphocyte count, 10^9^/L | 1.77 ± 0.66 | 1.40 ± 0.56 | 0.93 ± 0.35 | <0.001 |
| Monocyte count, 10^9^/L | 1.80 ± 12.71 | 0.50 ± 0.25 | 0.53 ± 0.30 | 0.413 |
| Hemoglobin, g/L | 125.79 ± 22.24 | 121.33 ± 25.90 | 118.56 ± 27.87 | 0.169 |
| RDW, % | 14.98 ± 12.41 | 13.76 ± 1.33 | 13.96 ± 1.38 | 0.491 |
| Platelet counts, 109/L | 182.30 ± 65.17 | 190.26 ± 62.67 | 189.18 ± 74.50 | 0.701 |
| ALT, IU/L | 30.08 ± 21.02 | 31.33 ± 35.04 | 98.55 ± 453.90 | 0.147 |
| Albumin, g/L | 39.84 ± 3.97 | 39.13 ± 4.31 | 37.82 ± 5.47 | 0.015 |
| Serum creatinine, umol/L | 97.93 ± 57.68 | 113.12 ± 89.03 | 162.70 ± 155.12 | <0.001 |
| LDL-C, IU/L | 2.46 ± 0.98 | 2.50 ± 1.07 | 2.48 ± 1.22 | 0.982 |
| Serum potassium, mmol/L | 4.07 ± 0.58 | 5.44 ± 14.71 | 4.13 ± 0.70 | 0.489 |
| Serum sodium, mmol/L | 138.65 ± 15.22 | 138.25 ± 14.79 | 138.39 ± 4.35 | 0.977 |
| Serum chlorine, mmol/L | 104.45 ± 4.39 | 103.66 ± 4.55 | 101.97 ± 5.61 | 0.003 |
| INR | 1.21 ± 0.32 | 1.18 ± 0.35 | 1.33 ± 0.68 | 0.103 |
| APTT, seconds | 39.45 ± 10.31 | 38.94 ± 7.10 | 42.79 ± 14.48 | 0.047 |
| PT, seconds | 14.98 ± 3.07 | 14.74 ± 3.39 | 16.10 ± 6.09 | 0.096 |
| NT-proBNP, pg/ml | 5371.44 ± 7787.35 | 6148.44 ± 7707.65 | 11334.22 ± 11302.40 | <0.001 |
| cTN-I, ng/ml | 0.12 ± 0.38 | 0.43 ± 1.31 | 2.06 ± 9.11 | 0.038 |
| LVEF, % | 48.80 ± 15.82 | 47.52 ± 15.05 | 47.64 ± 16.10 | 0.836 |
| **Comorbidities, n (%)** |  |  |  |  |
| Coronary atherosclerotic heart disease | 40 (45.98) | 49 (56.32) | 52 (59.77) | 0.165 |
| Atrial fibrillation | 45 (51.72) | 38 (43.68) | 36 (41.38) | 0.355 |
| Valvular disease | 53 (60.92) | 64 (73.56) | 70 (80.46) | 0.015 |
| Cardiomyopathy | 27 (31.03) | 35 (40.23) | 27 (31.03) | 0.336 |
| Chronic kidney disease | 13 (14.94) | 22 (25.29) | 39 (44.83) | <0.001 |
| Liver dysfunction | 6 (6.90) | 4 (4.60) | 4 (4.60) | 0.739 |
| Pneumonia | 13 (14.94) | 22 (25.29) | 36 (41.38) | <0.001 |
| Hypertension | 54 (62.07) | 67 (77.01) | 57 (65.52) | 0.086 |
| Diabetes | 30 (34.48) | 35 (40.23) | 35 (40.23) | 0.667 |
| Atherosclerosis | 79 (90.80) | 78 (89.66) | 80 (91.95) | 0.871 |

**Abbreviations:** SIRI: systemic inflammation response index. SBP: systolic blood pressure; DBP: diastolic blood pressure; SpO2: pulse oximetry-derived oxygen saturation; RDW: red cell volume distribution width; ALT: Alanine aminotransferase; LDL-C: Low-Density lipoprotein cholesterol; APTT: activated partial thromboplastin time; PT: prothrombin time; INR: international normalized ratio; NT-proBNP: N terminal pro B type natriuretic peptide; cTN-I: cardiac troponin I; LVEF: left ventricular ejection fraction;
