# Supplementary material for: Systemic Inflammation Response Index Is a Promising Prognostic Marker in Elderly Patients With Heart Failure: A Retrospective Cohort Study
Source: Front Cardiovasc Med. 2022 Jul 14;9:871031. doi: 10.3389/fcvm.2022.871031 (PMC9330028; doi:10.3389/fcvm.2022.871031)
Supplement: Supplementary file 3 [file Table_3.docx]

TableS3. Association between SIRI quintiles and clinical outcomes of in critically ill patients with congestive heart failure.

| **Clinical outcomes** | Model 1 | | Model 2 | | Model 3 | |
| --- | --- | --- | --- | --- | --- | --- |
|  | HR (95% CI) | *P value* | HR (95% CI) | *P value* | HR (95% CI) | *P value* |
| **Primary outcome** |  |  |  |  |  |  |
| 90-day all-cause mortality ^a^ |  |  |  |  |  |  |
| Q1 | 1.0 |  | 1.0 |  | 1.0 |  |
| Q2 | 1.13 (0.89, 1.43) | 0.3260 | 1.11 (0.87, 1.41) | 0.4005 | 1.11 (0.87, 1.41) | 0.4121 |
| Q3 | 1.31 (1.04, 1.65) | 0.0219 | 1.28 (1.02, 1.62) | 0.0369 | 1.20 (0.95, 1.52) | 0.1353 |
| Q4 | 1.46 (1.16, 1.83) | 0.0011 | 1.42 (1.13, 1.79) | 0.0024 | 1.29 (1.02, 1.63) | 0.0313 |
| Q5 | 1.95 (1.57, 2.42) | <0.0001 | 1.89 (1.52, 2.35) | <0.0001 | 1.48 (1.18, 1.86) | 0.0008 |
| *P for trend* |  | <0.0001 |  | <0.0001 |  | 0.0004 |
| **Secondary outcomes** |  |  |  |  |  |  |
| One-year all-cause mortality ^a^ |  |  |  |  |  |  |
| Q1 | 1.0 |  | 1.0 |  | 1.0 |  |
| Q2 | 1.01 (0.84, 1.22) | 0.8804 | 1.00 (0.83, 1.20) | 0.9910 | 0.99 (0.82, 1.19) | 0.9230 |
| Q3 | 1.16 (0.97, 1.39) | 0.1016 | 1.14 (0.95, 1.36) | 0.1546 | 1.06 (0.89, 1.28) | 0.5125 |
| Q4 | 1.19 (1.00, 1.43) | 0.0514 | 1.17 (0.98, 1.40) | 0.0855 | 1.05 (0.88, 1.27) | 0.5829 |
| Q5 | 1.60 (1.35, 1.90) | <0.0001 | 1.56 (1.32, 1.86) | <0.0001 | 1.26 (1.05, 1.51) | 0.0126 |
| *P for trend* |  | <0.0001 |  | <0.0001 |  | 0.0026 |
| Length of hospital stay ^b^ |  |  |  |  |  |  |
| Q1 | 0 |  | 0 |  | 0 |  |
| Q2 | 0.02 (-0.85, 0.89) | 0.9555 | -0.01 (-0.87, 0.85) | 0.9797 | -0.12 (-0.95, 0.70) | 0.7683 |
| Q3 | 0.33 (-0.54, 1.20) | 0.4592 | 0.21 (-0.65, 1.08) | 0.6279 | -0.17 (-1.00, 0.67) | 0.6984 |
| Q4 | 1.34 (0.47, 2.21) | 0.0026 | 1.18 (0.31, 2.05) | 0.0077 | 0.71 (-0.14, 1.56) | 0.1013 |
| Q5 | 2.45 (1.58, 3.32) | <0.0001 | 2.25 (1.37, 3.12 | <0.0001 | 0.97 (0.08, 1.86) | 0.0322 |
| *P for trend* |  | <0.0001 |  | <0.0001 |  | 0.0045 |
| Length of ICU stay ^b^ |  |  |  |  |  |  |
| Q1 | 0 |  | 0 |  | 0 |  |
| Q2 | 0.44 (-0.12, 1.01) | 0.1259 | 0.44 (-0.13, 1.00) | 0.1292 | 0.34 (-0.19, 0.86) | 0.2105 |
| Q3 | 0.65 (0.09, 1.22) | 0.0241 | 0.62 (0.05, 1.19) | 0.0333 | 0.30 (-0.23, 0.83) | 0.2679 |
| Q4 | 1.03 (0.46, 1.60) | 0.0004 | 0.98 (0.41, 1.55) | 0.0008 | 0.59 (0.05, 1.13) | 0.0334 |
| Q5 | 2.03 (1.46, 2.60) | <0.0001 | 1.96 (1.39, 2.53) | <0.0001 | 0.87 (0.31, 1.44) | 0.0025 |
| *P for trend* | 0.16 (0.12, 0.20) | <0.0001 | 0.15 (0.11, 0.19) | <0.0001 | 0.06 (0.02, 0.10) | 0.0034 |
| Renal replacement therapy ^c^ |  |  |  |  |  |  |
| Q1 | 1.0 |  | 1.0 |  | 1.0 |  |
| Q2 | 0.78 (0.56, 1.08) | 0.1341 | 0.77 (0.55, 1.07) | 0.1166 | 0.74 (0.52, 1.05) | 0.0896 |
| Q3 | 1.16 (0.85, 1.57) | 0.3489 | 1.13 (0.83, 1.53) | 0.4419 | 1.06 (0.76, 1.46) | 0.7428 |
| Q4 | 1.40 (1.04, 1.87) | 0.0261 | 1.35 (1.00, 1.82) | 0.0465 | 1.23 (0.90, 1.69) | 0.1991 |
| Q5 | 3.23 (2.47, 4.23) | <0.0001 | 3.11 (2.37, 4.08) | <0.0001 | 2.43 (1.80, 3.29) | <0.0001 |
| *P for trend* |  | <0.0001 |  | <0.0001 |  | <0.0001 |

**Abbreviations:** a: Cox proportional hazards regression models were used to calculate hazard ratios (HR) with 95% confidence intervals (CI). b: Linear regression model were used to calculate β value with 95% confidence intervals (CI). c: Logistic regression models were used to calculate odds ratios (OR) with 95% confidence intervals (CI).

Model 1 covariates were adjusted for nothing; Model 2 covariates were adjusted for age, sex and ethnicity; Model 3: a: covariates were adjusted for age, sex, ethnicity, systolic blood pressure, diastolic blood pressure, system inflammatory response syndrome, serum creatinine, hemoglobin, white blood cell count, platelet count, red cell volume distribution width, atrial fibrillation, coronary artery disease, chronic kidney disease, respiratory failure, pneumonia, hypertension; b: covariates were adjusted for age, sex, ethnicity, blood urea nitrogen, white blood cell count, platelet count, atrial fibrillation, respiratory failure, pneumonia, SOFA, SASP II; c: covariates were adjusted for age, sex, ethnicity, diastolic blood pressure, respiratory rate, anion gap, serum creatinine, white blood cell count, platelet count, red cell volume distribution width, atrial fibrillation, chronic kidney disease, respiratory failure, pneumonia, SOFA, SASP II.
